# The strategies that peanut and nut-allergic consumers employ to remain safe when travelling abroad

**DOI:** 10.1186/2045-7022-2-12

**Published:** 2012-07-09

**Authors:** Julie Barnett, Neil Botting, M Hazel Gowland, Jane S Lucas

**Affiliations:** 1 Brunel University, London, UK; 2 Academic Unit of Clinical and Experimental Sciences, Faculty of Medicine, University of Southampton, Southampton, UK; 3 Allergy Action, London, UK; 4 Mailpoint 803. Clinical and Experimental Sciences, Faculty of Medicine, University Hospital Southampton NHS Foundation Trust, Tremona Road, Southampton, UK , SO16 6YD

**Keywords:** Food allergy, Peanut allergy, Tree nut allergy, Holiday, Travel, Airline

## Abstract

**Background:**

An understanding of the management strategies used by food allergic individuals is needed as a prerequisite to improving avoidance and enhancing quality of life. Travel abroad is a high risk time for severe and fatal food allergic reactions, but there is paucity of research concerning foreign travel. This study is the first to investigate the experiences of, and strategies used by peanut and tree nut allergic individuals when travelling abroad.

**Methods:**

Thirty-two adults with a clinical history of reaction to peanuts or tree nuts consistent with IgE-mediated allergy participated in a qualitative interview study.

**Results:**

Travel abroad was considered difficult with inherent risks for allergic individuals. Many participants recounted difficulties with airlines or restaurants. Inconsistency in managing allergen avoidance by airlines was a particular risk and a cause of frustration to participants. Individuals used a variety of strategies to remain safe including visiting familiar environments, limiting their activities, carrying allergy information cards in the host language, preparing their own food and staying close to medical facilities.

**Conclusions:**

Participants used a variety of allergen avoidance strategies, which were mostly extensions or modifications of the strategies that they use when eating at home or eating-out in the UK. The extended strategies reflected their recognition of enhanced risk during travel abroad. Their risk assessments and actions were generally well informed and appropriate. A need for airline policy regarding allergy to be declared and adhered to is needed, as is more research to quantify the true risks of airborne allergens in the cabin. Recommendations arising from our study are presented.

## Introduction

Food allergy is an important health problem, with an estimated 1% of the population having an IgE mediated food allergy, and evidence that the prevalence continues to increase [1,2]. Food allergy cannot be cured and management is therefore focused on allergen avoidance and prompt treatment of serious reactions [3]. Morbidity is usually low and food allergy is rarely fatal, with 48 deaths in the UK between 1999 and 2006 [4]. However, the need for constant vigilance to avoid particular food allergens [5,6], the need to carry adrenaline auto-injectors [7,8] and the fear of anaphylaxis, all impact on food allergic individual’s social life, emotional wellbeing and quality of life [9]. Food is central to many social situations and a variety of strategies are needed to avoid allergens in different settings. For example the methods used to avoid allergens when shopping for one’s self [5,10] are different to those used when eating in a restaurant [6], or attending a family celebration. Travelling abroad is another situation where food allergic individuals may encounter particular difficulties given that mistakes are more likely to occur in unfamiliar situations [4,11].

A literature search via Ovid Medline using the terms travel*, holiday vacation and *abroad* combined with allergy, food hypersensitivity, adverse reactions, nut allergy, food allergy, quality of life, psycho* and *psychosocial,* identified a paucity in the literature regarding travelling and allergy. Most papers focused on difficulties in-flight [12-15]. Importantly, in one study 9% of participants reported adverse reactions on aircraft, 80% of which were moderate or severe [12]. Another study suggested that only 38% of patients who requested special consideration from an airline due to food allergy actually received satisfactory assistance [13]. The difficulties of air travel for food allergic individuals, are confirmed by a survey of in-flight paediatric medical emergencies which reported that 9% were due to allergic reactions [15]. Besides surveys focusing on air travel, little has been reported about the difficulties of travelling abroad with a peanut or tree nut allergy. Physicians, dieticians and other health professionals have a vital role in advising patients how to avoid allergens [16] but this requires an understanding of allergic patients’ current behaviour. Qualitative research methods, for example in-depth interviews, provide a powerful means to gain depth of understanding of patients’ experiences and perspectives regarding their allergies [17]. This study is the first detailed report on the experiences, challenges and decisions made by peanut and tree nut allergic individuals when considering travelling at home and abroad. The study was designed to provide insights into participants’ previous experiences and how these informed behaviour.

## Methods

Ethical approval was gained from the National Research Ethics Service and the University of Surrey Ethics Committee (approval number 09/H1109/64). The aim of the research program was to understand the complexities and reasoning behind decisions made by food allergic individuals when purchasing and consuming food. This manuscript focuses on the data relating to travel abroad.

### Study population

The study population and methods used for the in-depth interviews study have previously been published in detail [5,6]. In brief, thirty-two volunteers were recruited from three sources to ensure diversity of allergy profile and experience; University Southampton Hospital (USH) specialist allergy clinics, GP surgeries and an email advert to staff and students of University of Surrey. All participants reported a doctor diagnosis of IgE mediated allergy to peanuts or tree nuts (from now on jointly referred to as nut allergic individuals). A history of systemic allergic reaction to foods other than peanuts or tree nuts excluded the volunteer from recruitment to this study. Volunteers from the University of Surrey and from primary care settings reported being seen by their GP or a hospital specialist who had diagnosed peanut or tree nut allergy and prescribed rescue medication. Participants were required to be 16 years old or above and were fluent in the English language. Twenty-two individuals who were approached were eligible, but declined to be involved in the study, resulting in a 59.3% response rate. The severity of a participant’s worst ever reaction to nuts was graded using a classification previously used for peanut allergy [18].

### Data collection

Subjects were assessed for eligibility by a screening questionnaire. Eligible subjects participated in in-depth semi-structured interviews conducted in their home by a researcher experienced in qualitative interviews. Participants were specifically asked about previous experiences when travelling abroad and how they make decisions regarding food choices when eating abroad. The interview was audio recorded and transcribed verbatim.

### Data analysis

Thematic coding [19] (NVIVO qualitative data analysis software; version 8 2008; QSR International Pty Ltd) was used to capture the key points and opinions pertaining to travel. Interpretations were developed looking at both converging and diverging views within the themes. Follow up interviews provided the opportunity to confirm the relevance of the codes and analysis. Quality was assured by the research team regularly checking the interpretations of the lead analyst (J.B) and confirming their validity. Literal transcriptions of selected relevant answers are shown in (Additional files 1, 2, 3, 4 and 5: Boxes 1, 2, 3, 4 and 5).

## Results

Characteristics of participants have previously been described [5,6]. Briefly, thirty-two participants, who had previously been prescribed emergency treatment to be carried in case of allergic reactions, were interviewed. Their ages varied from 16–70 years (median 31 years). There were nine males and twenty-three females. Twenty-two participants were recruited from a specialist allergy clinic. Five participants had peanut allergy, nine tree nut allergy and eighteen peanut plus tree nut allergy. Two participants had previously experienced mild reactions only, twelve moderate and eighteen severe.

The overarching theme described by participants was simply that it was difficult to travel abroad; foreign holidays were seen to pose a range of additional and necessary considerations, in comparison to staying in UK. Answers to questions about bad experiences were most instructive as a good experience was usually viewed in terms of the absence of a bad one.

The main themes which emerged were: choice of destination, issues with air travel, accessibility to medical care, issues around unfamiliarity of the destination and avoidance of high risk foods. Examples of participants’ quotes to demonstrate these themes are presented in Additional files 1, 2, 3, 4 and 5: Boxes 1, 2, 3, 4 and 5.

### Destination choice

Many participants discussed their nut allergy as a key determinant of their choice of holiday destination (Additional file 1: Box 1: Quote A). Some participants only considered going abroad to English speaking countries, or countries where the participants perceived less of a language barrier (Additional file 1: Box 1: Quotes B and C). A language barrier was perceived to have less impact if the individual had a partial understanding of the foreign language or if the destination country was English-speaking (Additional file 1: Box 1: Quote B). Some participants specifically avoided destinations where the local cuisine was perceived as high risk. For example, Asia was considered problematic, due to the perception that food in countries such as China contains nuts or peanuts (Additional file 1: Box 1: Quote D). Conversely, familiar cuisine encouraged some nut allergic individuals to travel to a country, for example one participant felt relatively confident travelling to Italy because they frequently eat Italian food in the UK without adverse effects (Additional file 1: Box 1: Quote C).

Food allergy also affected the *type* of holiday nut allergic individuals chose. For example participants expressed a preference for self–catering holidays so that they had a greater control of their diets. They also avoided certain types of holiday which could place them at unnecessarily high risk (Additional file 1: Box 1: Quote B,E).

At the extreme end of the spectrum, some people did not go abroad on holiday at all, sticking to UK destinations in order to prevent adverse reactions to food (Additional file 1: Box 1: Quote F).

### Issues with air travel

Many participants highlighted air travel as an issue when going on holiday abroad, with participants experiencing both positive and negative experiences. There was a discrepancy between different airlines in terms of how ground and air staff treated nut allergic individuals. Positive experience with a particular airline would be more likely to result in repeat custom and greater ease of mind (Additional file 2: Box 2: Quote A). Examples of perceived good airline practice reported by participants included not selling peanuts, making announcements to stop other passengers eating nuts and ‘keeping an eye out’ for a nut allergic individual (Additional file 2: Box 2: Quotes B and C). These all contributed to the individual feeling safe whilst in-flight. In some cases, air stewards approached the nut allergic individuals and asked their permission to hand out nut-based snacks, prior to doing so (Additional file 2: Box 2: Quote D). Conversely, some people had bad experiences on aeroplanes, including being served nuts despite making it clear to the air staff that they were allergic (Additional file 2: Box 2: Quote C).

Some participants were reluctant to disclose their allergy to airlines in-case of an over-cautious response, such as being offered a poorer selection of food on a long haul flight (Additional file 2: Box 2: Quote E). Indeed, the length of the flight altered the habits of nut allergic individuals. On a short haul flight, they were more likely to take their own food and not eat the food provided (Additional file 2: Box 2: Quote F). Some participants took a pragmatic approach to flying by waiting to see what was served on the flight and only eating the food taken from home if necessary (Additional file 2: Box 2: Quote G).

### Accessibility of medical care

The availability of medical care or remoteness of their location when abroad influenced participant choices of destinations and behaviour whilst on holiday. Several participants described how they were willing to take more ‘risks’ in situations where they were closer to immediate medical care (e.g. in cities) than in situations where they were in a remote location (Additional file 3: Box 3: Quote A). In some cases, the nut allergic individual would not engage in activities which would take them to remote locations where medical assistance is hard to access. For example, one participant felt unable to accompany her husband on an expedition to Kenya, purely because of her food allergy; being hours away from medical care was considered too much of a risk (Additional file 3: Box 3: Quote B). Some participants expressed regret that their allergy restricted them doing spontaneous things when in remote locations, due to the fear of an adverse reaction (Additional files 3: Box 3: Quote C). Planning played a large part in ensuring a safe holiday and this included knowing where the nearest hospital was and how to get there in case of an emergency (Additional file 3: Box 3: Quote D).

### Familiarity

Travelling to unfamiliar destinations posed problems for nut allergic individuals because they did not know what to expect in terms of managing their allergies. Many participants returned to familiar destinations where they had had positive experiences and where they trusted those managing the food preparation (Additional file 4: Box 4: Quote A) and some would not be comfortable travelling to new, unfamiliar countries (Additional file 4: Box 4: Quote B).

For many people, unfamiliarity did not prevent them from travelling to a foreign country. However, they took precautions to make the experience safer. One recurring method involved the use of translation cards with various statements in the relevant foreign language. These cards might include statements such as “I have a nut allergy. Can you cater for me? I am allergic to ……….” or “My child is having an anaphylactic shock. Could you ring the ambulance?”. These were considered useful when eating in restaurants and provided peace of mind (Additional file 4: Box 4: Quote C). Some participants simply learnt the word for ‘peanut’ so they could read menus or supermarket ingredients and work out if something is safe to eat (Additional file 4: Box 4: Quote D). Most people did this even if additionally purchasing translation cards. Due to the cost of translation cards, some people elected to make their own (Additional file 4: Box 4: Quote E).

Besides learning rudimentary foreign words and using translation cards, nut allergic individuals took other precautions when travelling to unfamiliar places. For example, one participant stated she normally carries two auto-injectors, but when travelling she’ll carry six, in case she is unable to get medical care promptly after a reaction (Additional file 4: Box 4: Quote F). She goes on to say how she takes a comprehensive first aid kit whilst abroad due to the risk of having an allergic reaction. A further precaution discussed during the interviews was the use of Medic Alert bracelets to make the exact allergy clear and provide a telephone number for emergency instructions (Additional file 4: Box 4: Quote G).

### Avoidance of high risk foods

Frequently participants implemented avoidance strategies similar to those used at home. However, these needed modifying due to the unfamiliar surroundings and language barriers. Several people stated that they restricted themselves to eating plain foods, for example simple steak, and would turn down foreign cuisine which they haven’t eaten before or were not confident of being nut-free (Additional file 5: Box 5: Quotes A and B). Some participants recognised this was ‘boring’ but was the safest strategy, particularly when eating in restaurants (Additional file 5: Box 5: Quote C; Box 3 Quote C) and would only eat in restaurants where the cuisine was familiar (Additional file 5: Box 5: Quote D). More specifically, participants were inclined to avoid desserts when eating out in a foreign country, due to the perception that desserts are more likely to contain nuts (Additional file 5: Box 5: Quote E).

Being abroad and not understanding labelling practices was problematic for some nut allergic individuals when buying food from supermarkets (Additional file 5: Box 5: Quote F). Others found this less of a problem and believed that they merely needed to learn the word for ‘peanut’ to shop safely. Indeed, solely buying food in supermarkets to prepare for themselves was a strategy some participants decided to adopt (Additional file 5: Box 5: Quote G).

These results demonstrate the range of diverse strategies that people used in an attempt to remain safe. However, there was a sense of tension between the desires to dispense with inhibitions on holiday, whilst having to avoid allergen exposure. One participant clearly articulated the lack of spontaneity that resulted from the constant need to remain safe (Additional file 5: Box 3: Quote C), and another commented that her diet on holiday seemed boring, but safe (Additional file 5: Box 5: Quote C).

## Discussion

This is the first study to make a detailed analysis of the strategies that peanut and nut allergic individuals use to remain safe when travelling abroad. Although all participants considered foreign travel difficult, they varied in how they dealt with this. Some individuals simply decided to consider foreign travel too much of a risk, and always holidayed locally. However, most took holidays abroad, but planned carefully with self-imposed restrictions to reduce risk.

The strategies that participants used to remain safe were generally well thought through, and were mostly extensions or modifications of those used at home. For example when on holiday, they would choose food and restaurants using similar principles to those used at home [6], and they were particularly cautious of risks in remote locations. However, there were some, perhaps more risky strategies that were frequently mentioned during the interviews when discussing eating in UK [5,6,10], that were not discussed in relation to eating abroad. For example, when eating at home or in UK restaurants, participants had frequently described relying on sensory evidence such as taste, the look of the product and the smell to help them decide whether to eat a food. This was not described by participants when discussing eating abroad. The more cautious approach, may explain the disappointment described by some around the lack of possibilities for spontaneity and the requirement for everything to be planned.

Many participants described how their food allergy played a key role in selecting a holiday destination and type of holiday. For example, participants might not travel to a country where the cuisine was unfamiliar or used a lot of nuts, thus reducing unnecessary risk from the outset. Specifically, access to medical care impacted on destination choice, but also on behaviour whilst abroad. Despite their allergies, many participants still enjoy travelling and would visit new, unfamiliar places. In addition to avoidance strategies, many introduced tools to help them remain safe, for example using translation cards or carrying extra medication.

The majority of literature concerning travel has focused on air- travel and this was certainly an important consideration for our participants. They described both positive and negative experiences on aircraft. They considered good airline practice to include making flights ‘nut-free’ or even upgrading nut allergic individuals if nuts were being served in the cabin. However, some participants described scenarios when they have been served nuts despite telling the cabin crew they were allergic. Participants developed strategies to deal with this, in particular taking their own food on the flight, but other factors came into play when determining what precautions to put in place, such as the length of the flight and previous experiences whilst flying with the airline. Specific behaviour by people who have had a previous reaction whilst flying have been described. Greenhawt et al reported that 52% of allergic individuals would change their flying behaviour, with 25% no longer consuming airline food, 24% cleaning their seating area, 20% requesting a nut-free flight and 12% no longer flying [13]. Other than cleaning the seating area, these were all behaviours described in our study. Comstock et al investigated the mode of exposure that allergic individuals reported having triggered a reaction [12]. Fifty eight percent reported exposure by inhalation, 33% by ingestion and 9% by direct contact. The high number of reactions associated with inhalation of food allergens suggests that nut-free flights are necessary to prevent in-flight reactions entirely. As food allergens persist in the environment, restrictions might be necessary on all flights and not only those on which nut allergic individuals are travelling. However, it should be noted that in a study of patients who reported reactions to inhalations of peanut, participants did not react on blinded inhalational challenges [20].

The heightened precautions taken by nut allergic individuals whilst travelling abroad seem entirely appropriate in the light of mortality figures, concerns about language barriers, unfamiliarity with foods and the high incidence of nuts in some cuisines. Published UK data of fatal allergic reactions between 1999 and 2006, reported that 4 out of 48 fatalities occurred in a foreign country [4]. This is supported by unpublished data of fatal allergic reactions to foods known to UK Anaphylaxis Campaign over the past 20 years; of 139 fatal reactions, 14 occurred in British citizens whilst abroad and 4/139 were overseas visitors to the UK. (Personal communication, Hazel Gowland). The strategies used by participants in this study support previous US data which described careful selection of destination (68%), avoidance of specific countries due to allergy, packing extra medication (67%), packing suitable foods (94%) and discovering where the closest hospital is to their destination (48%) [21].

Lack of familiarity with labelling regulations in different countries is relevant, but was mentioned surprisingly little by participants, possibly reflecting their lack of awareness of this issue although it may be the case that it was deemed irrelevant insofar as pack labelling would not have been understood anyway. Indeed, we have recently reported that these participants were unaware of laws and regulations surrounding labelling that are applicable in the UK [5,10]. Following an EU-wide review of labeling legislation, in 2011 the European Parliament approved the Food Information for Consumers Regulation (FIR) (EU 1169/2011). This will provide greater consistency of packaged food labelling practice across the European Union in terms of multi-lingual wording, minimum text sizes and greater responsibility of manufacturers to provide information for consumers.

It will also require catering services provided by transport services to label foods that contain one of the fourteen main food allergens as an ingredient, but does not address the declaration of possible allergen cross contamination. The FIR came into effect after this study ended. It should be noted that it only applies where the journey starts within a member state. Policies now need to be implemented to ensure that the implications of this regulation are understood, particularly since it does not apply to return flights that start outside Europe. The study focused on individuals with peanut and tree nut allergy, but similar problems will be encountered by people with allergies to other foods. As part of the study design, we recruited subjects from a variety of settings, including a specialist allergy centre and a mail shot to University staff and students, with the aim of increasing the diversity of the study population. However, it is likely that as research volunteers, they were highly motivated individuals and perhaps better informed than some others.

### Conclusions and implications

Travelling abroad is a high risk situation for individuals with food allergies. Our study demonstrates that nut allergic individuals use a range of strategies to minimise the risks, primarily based on patterns that they implement at home. We have highlighted a number of situations that are particularly problematic for nut allergic individuals during travel. Airline flights are of particular concern for individuals and this is exacerbated by inconsistent information by airlines and their staff. This study demonstrates that nut allergic individuals are taking sensible steps to remain safe, and it is now time for the travel industry to take responsibility for the safety of their customers and develop a consistent approach to allergic individuals (Table 1). Healthcare professionals should be able to advise their patients and direct them to reliable sources of information for travellers with allergy (Table 2). All staff responsible for preparing or serving food, whether working in restaurants, other food outlets or on transport require training in order to keep food allergic individuals safe. Research is also needed to understand the true ‘cabin risk’ caused by airborne allergens.

**Table 1:** Recommendations arising from this study


1.	International policy needs to be developed to provide allergy information in catering services provided by transport e.g. planes and trains. This policy should cover the use of the 14 main allergens as ingredients **and** cross contamination.
2.	Policy should be developed on how to manage the increased risk of food allergic individuals when eating in confined spaces e.g. airline cabin.
3.	International regulations should address the training of all staff responsible for preparing and serving food in transport services, restaurants and other food outlets.
4.	Further research is required to quantify the true risk of airborne allergens in aircraft cabins.

**Table 2:** How can the Health professional respond to frequently asked questions?


I have informed the airline that I have peanut allergy, will my meal be safe?	Many airlines will provide a special meal on request. Check that the information has been passed on at every possible interaction you have with the airline. Ask when checking in, when boarding and when given the meal. For complete reassurance some allergic individuals prefer to provide their own meal.
What if previous passengers have been eating peanuts whilst sitting in my seat?	The most likely reaction will be due to skin contact. The risk can be reduced by carrying wipes to clean any hard surfaces as soon as you board.
Am I at risk of inhalation reactions through peanut in the atmosphere?	The risk of inhalation reactions is controversial, but is probably low. If your doctor considers you at high risk (e.g. poorly controlled asthma or previous inhalation reactions), you may wish to contact the airline at the time of booking to establish whether the flight can be made ‘nut free’. However, allergens may persist from previous flights, and the request may limit your choice of airlines.
Where can I find information for allergic individuals about travelling abroad?	Check your airline website for their policies. General advice can be found at the following websites:
	http://www.anaphylaxis.org.uk/living-with-anaphylaxis/travel
	http://www.allergyaction.org/allergy_action1.htm
	http://www.iata.org
Can I carry my allergy auto-injector on the flight?	**YES,** you should carry it at all times. Also carry your emergency action plan provided by your medical team. Contact the airline in advance to ask their advice on carrying the auto-injector. Approach security to inform them that you are carrying the auto-injector for medical purposes. Advise the cabin crew that you have it, and let them know where to find it. You may also like to wear an emergency alert bracelet
How can I eat safely if I don’t understand the language?	Obtain translations of key words and sentences before travel. Perhaps take cards with images of foods to be avoided. Familiarise yourself with the cultural diet in advance and work out what you need to avoid. If you are going to a country with complex, high-risk cuisine, self-catering may be the safest option. If you are considering taking foods with you, check in advance with the airline whether this is permissible, and whether your destination country will allow you to take food in.
Can I obtain translations to let people know about my allergies?	Yes the following website has translations of key phrases in a number of languages:
	http://www.allergyaction.org/allergy_action1.htm

## Competing interests

The authors declare that they have no competing interests.

## Authors’ contributions

All authors participated in the preparation of the manuscript and all approved the final manuscript. JB, MHG and JSL participated in the study design. JB was principal investigator with primary responsibility for the study, supervision of interviews and analysis of data. MHG provided advice as an allergic consumer. NB analysed the data pertinent to this publication and drafted the text. JSL provided clinical expertise for the study design and analysis. JSL led the writing of this manuscript.

## Authors’ information

**Julie Barnett** is Reader in Healthcare Research at Brunel University; she is a social psychologist with particular research interests in the way in which people understand and communicate risk information, in behaviour change and in relation to how and when to engage and involve people in the development of policy and practice. **Neil Botting** is a Medical Student at University of Southampton. **Hazel Gowland** has been allergic to nuts and peanuts since 1960 and has survived a number of life-threatening reactions. She has worked at national level with the Anaphylaxis Campaign since its earliest days in 1994 and is now its Food Adviser. HG supports and advises those at risk from severe food allergies, both through personal experience and professional expertise. This work involves food suppliers as well as families, schools, food enforcement officers, local and national government, doctors, specialist nurses and dieticians. **Jane S Lucas** is an allergist at University Hospital Southampton NHS Foundation Trust and a Senior Lecturer at University of Southampton.

## Supplementary Material

Additional file 1 **Box 1.** Destination Choice. Click here for file

Additional file 2 **Box 2.** Issues with air travel. Click here for file

Additional file 3 **Box 3.** Accessibility of medical care. Click here for file

Additional file 4 **Box 4.** Unfamiliarity. Click here for file

Additional file 5 **Box 5.** Avoidance of high risk foods. Click here for file

## References

[B1] Grundy J Matthews S Bateman B Dean T Arshad SH Rising prevalence of allergy to peanut in children: data from 2 sequential cohorts J Allergy Clin Immunol 2002 110 784 789 10.1067/mai.2002.128802 12417889

[B2] Gupta R Sheikh A Strachan DP Anderson HR Time trends in allergic disorders in the UK Thorax 2007 62 91 96 10.1136/thx.2004.038844 16950836 PMC2111268

[B3] Simons FE Emergency treatment of anaphylaxis BMJ 2008 336 1141 1142 10.1136/bmj.39547.452153.80 18497373 PMC2394586

[B4] Pumphrey RS Gowland MH Further fatal allergic reactions to food in the United Kingdom, 1999–2006 J Allergy Clin Immunol 2007 119 1018 1019 10.1016/j.jaci.2007.01.021 17349682

[B5] Barnett J Leftwich J Muncer K Grimshaw K Shepherd R Raats MM Gowland MH Lucas JS How do peanut and nut-allergic consumers use information on the packaging to avoid allergens? Allergy 2011 66 969 978 10.1111/j.1398-9995.2011.02563.x 21320134

[B6] Leftwich J Barnett J Muncer K Shepherd R Raats MM Hazel GM Lucas JS The challenges for nut-allergic consumers of eating out Clin Exp Allergy 2011 41 243 249 10.1111/j.1365-2222.2010.03649.x 21121977

[B7] Macadam C Barnett J Roberts G Stiefel G King R Erlewyn-Lajeunesse M Holloway JA Lucas JS What factors affect the carriage of epinephrine auto-injectors by teenagers? Clin Transl Allergy 2012 2 3 10.1186/2045-7022-2-3 22409884 PMC3299626

[B8] Gallagher M Worth A Cunningham-Burley S Sheikh A Epinephrine auto-injector use in adolescents at risk of anaphylaxis: a qualitative study in Scotland, UK Clin Exp Allergy 2011 41 869 877 10.1111/j.1365-2222.2011.03743.x 21481022

[B9] Cummings AJ Knibb RC King RM Lucas JS The psychosocial impact of food allergy and food hypersensitivity in children, adolescents and their families: a review Allergy 2010 65 933 945 10.1111/j.1398-9995.2010.02342.x 20180792

[B10] Barnett J Muncer K Leftwich J Shepherd R Raats MM Gowland MH Grimshaw K Lucas JS Using 'may contain' labelling to inform food choice: a qualitative study of nut allergic consumers BMC Publ Health 2011 11 734 742 10.1186/1471-2458-11-734 PMC3195759 21943285

[B11] Sicherer SH Furlong TJ Munoz-Furlong A Burks AW Sampson HA A voluntary registry for peanut and tree nut allergy: characteristics of the first 5149 registrants J Allergy Clin Immunol 2001 108 128 132 10.1067/mai.2001.115755 11447394

[B12] Comstock SS DeMera R Vega LC Boren EJ Deane S Haapanen LAD Teuber SS Allergic reactions to peanuts, tree nuts, and seeds aboard commercial airliners Ann Allergy Asthma Immunol 2008 101 51 56 10.1016/S1081-1206(10)60835-6 18681085

[B13] Greenhawt MJ McMorris MS Furlong TJ Self-reported allergic reactions to peanut and tree nuts occurring on commercial airlines J Allergy Clin Immunol 2009 124 3 599 600 10.1016/j.jaci.2009.06.039 19733300

[B14] Sicherer SH Furlong TJ DeSimone J Sampson HA Self-reported allergic reactions to peanut on commercial airliners J Allergy Clin Immunol 1999 104 186 189 10.1016/S0091-6749(99)70133-8 10400859

[B15] Moore BR Ping JM Claypool DW Pediatric Emergencies on a US-Based Commercial Airline Pediatr Emerg Care 2005 21 725 729 10.1097/01.pec.0000186424.84764.94 16280945

[B16] Clark AT Ewan PW Good prognosis, clinical features, and circumstances of peanut and tree nut reactions in children treated by a specialist allergy center J Allergy Clin Immunol 2008 122 286 289 10.1016/j.jaci.2008.05.015 18586319

[B17] Gallagher M Worth A Sheikh A Clinical allergy has much to gain from engagement with qualitative research Allergy 2009 64 1117 1119 10.1111/j.1398-9995.2009.02065.x 19432935

[B18] Hourihane JO Kilburn SA Dean P Warner JO Clinical characteristics of peanut allergy Clin Exp Allergy 1997 27 634 639 10.1111/j.1365-2222.1997.tb01190.x 9208183

[B19] Braun V Clarke V Using thematic analysis in psychology Qual Res Psychol 2010 3 77 101

[B20] Simonte SJ Ma S Mofidi S Sicherer SH Relevance of casual contact with peanut butter in children with peanut allergy J Allergy Clin Immunol 2003 112 1 180 182 10.1067/mai.2003.1486 12847496

[B21] Leonard S Weiss C Furlong T Sicherer S Food allergies affect vacation planning J Allergy Clin Immunol 2009 123 S28

