# Supplementary material for: The strategies that peanut and nut-allergic consumers employ to remain safe when travelling abroad
Source: Clin Transl Allergy. 2012 Jul 9;2:12. doi: 10.1186/2045-7022-2-12 (PMC3480958; doi:10.1186/2045-7022-2-12)
Supplement: Additional file 1 — Box 1. Destination Choice. [file 2045-7022-2-12-S1.docx]

Box 1: Destination Choice

| A | *“Yeah, it’s amazing that it can affect in that way, even in the way that you then go and make choices about things like where you’re flying to and… Correct, holiday destinations*. *Yeah. So we’re thinking, this summer, we’re thinking shall we go to Corfu, shall we go to Crete, and we had the map out of the [Airline X] flights seeing where they were going to, where they’re going to, to make our decision on where we would actually go.” [1069, M, Severe]* |
| --- | --- |
| B | *“Then, whenever we go away on a proper holiday, we always go to either English speaking or Spanish speaking countries because they’re the languages we can speak, and if we’re on our own, so not with family, we always go self-catering, and so then we just read the ingredients on everything that we buy, so it’s the same as here, yeah.*” *[1003, F, Mild]* |
| C | “*Em…well, I guess when I’m abroad on holiday, I don’t….I guess… We’ve mainly been on holiday to sort of Italy and America. So America isn’t a problem, because there’s no language barrier and I just ask and will sort of eat whatever. In Italy, I feel a lot safer, because I think I, you know, know Italian food quite well, so pizzas and pastas are generally okay.” [1116, F, Severe]* |
| D | *“And Asia as well – I mean, I’d love to go to China and I’d love to go to India as well, but there’s no way that it’s…it’s worth the risk really, so…” [1116, F, Severe]* |
| E | *“I wanted to go on a walking holiday, partly in Marrakech. The walking, it’s in the middle of nowhere, and you know, the guide would cook for you in the evening. I wrote – I knew that Moroccan cuisine had tons of nuts in, very nut-based indeed. I wrote to the company and said, you know, I’m allergic to nuts – do you think it’s realistic for me to, you know, to go on this holiday, given my nut allergy, and they said that, you know, unless you speak really fluent French, people won’t… They said people won’t understand what it is, and secondly, you won’t be able to explain it, so people won’t…you know, people won’t respond.” [1068, F, Moderate]* |
| F | *“We never go abroad….. We just go round the UK. We haven’t been brave enough to….. And why haven’t we gone to Europe? Exactly that reason: how do we know what the foods are going to be like when we get there?” [1069, M, Severe]* |

**Key:** The study ID number is followed by the patient gender (F stands for female and M for male), followed by the severity of the participant’s worst allergic reaction to peanuts or tree nuts.
