# Supplementary material for: The strategies that peanut and nut-allergic consumers employ to remain safe when travelling abroad
Source: Clin Transl Allergy. 2012 Jul 9;2:12. doi: 10.1186/2045-7022-2-12 (PMC3480958; doi:10.1186/2045-7022-2-12)
Supplement: Additional file 2 — Box 2. Issues with air travel. [file 2045-7022-2-12-S2.docx]

Box 2. Issues with air travel

| A | *“We go with [name of airline] and they’ve only got one aeroplane, and so they’re…that sort of level of service and attention to detail, they’ll do that, and for us, that’s important. If we want to go anywhere on a plane, we sort of see where they’re flying to. We know, when we say we’ve got this problem, they’ll definitely do something about it.” [1069, M, Severe]* |
| --- | --- |
| B | *“We go on holiday, as a family for instance, on aeroplanes. We speak to the airline before we go and ask them to make it a no-nut flight. They’ve got a choice of nibbles that they can give out to people – like nuts or cheesy bits. Well, give them the cheese biccies, not the nutty ones please. Well, the thing is, you’re in a confined sort of cylinder for four or five hours, with this air re-circulating. Giving out nuts is like…it’s terrible!” [1069, M, Severe]* |
| C | *“[Name of airline] is brilliant, absolutely brilliant! They will make an announcement – they ask you where you’re sitting, and they make an announcement saying, you know, please do not eat peanuts on this flight. They won’t sell any peanuts and, you know, they sort of keep an eye on you a bit, which is good. Who was bad? [Name of airline] were terrible – absolutely terrible! In fact, I told the stewardess that I had a nut allergy – I didn’t say peanut, I just said nut allergy – and her reaction was to stroke my arm and say, “Poor you,” and I had to explain to her, you know, “I’m telling you because you can’t give me nuts on this flight, and people shouldn’t eat nuts around me.” She was like, “Oh, okay, yeah, yeah – we don’t serve nuts.” I said, “Also, I have two adrenalin injections in my bag, which I thought you might like to know,” and she was like, “Oh okay,” and then, an hour later, served me a salad that had walnuts over the top.” [1116, F, Severe]* |
| D | *“I tell the air stewardess or air steward that I’m carrying [name of adrenaline auto-injector] for a nut allergy, and I have had two airlines come to me, in advance of serving any food, saying, “We’re going to hand out Macadamia nuts,” or whatever nuts they’re going to hand out. “If you have a problem with that, we won’t do it”……….. I think it’s excellent they do it, because obviously somebody could be breathing it in, if someone’s highly, highly sensitive, they need to do that.” [1029, F Severe]* |
| E | *“And also, the other thing is, if you’re not careful, if you tell people you’re allergic to nuts, you end up getting all the crap meals because they get so – like on a plane, if you tell them that you’ve got a nut allergy, they just end up giving you fresh fruit, whereas your partner’s got this really gorgeous chocolate cake, which you can eat because it’s got no nuts in, so that always really winds me up.” [3024, F, Severe]* |
| F | *“Yeah. aeroplane food is dreadful, and every carrier is different. …….. [Name of airline] are mediocre on the whole thing. And it depends how long haul I’m going. If it’s just going across to Europe, I’ll just go, oh, forget it, I’m not going to bother eating, and I’ll buy my own sandwiches or make my own, or just think I won’t bother to eat, I’ll sleep. When I’m going to Australia, I’ll fly [Name of airline] this time. They’re really good, and they have a no nut allergy thing. [Name of airline] is a pain in the backside, because you can have every single thing possible wrong with you, except nut allergy. They will not recognise it. So if you said you wanted to eat vegetarian food, they could give you nut food. The only thing you could have is a fruit platter………For 24 hours, all you eat is fruit.” [4013, F, Severe]* |
| G | *“I will take my own food on planes, because… I mean, sometimes I eat the plane food. I wait to see what they serve up. Some things are really obvious and, you know, you can tell they don’t have nuts in – well, I guess you think you can tell [laughing].” [1116, F, Severe]* |

**Key:** The study ID number is followed by the patient gender (F stands for female and M for male), followed by the severity of the participant’s worst allergic reaction to peanuts or tree nuts.
