# Supplementary material for: The strategies that peanut and nut-allergic consumers employ to remain safe when travelling abroad
Source: Clin Transl Allergy. 2012 Jul 9;2:12. doi: 10.1186/2045-7022-2-12 (PMC3480958; doi:10.1186/2045-7022-2-12)
Supplement: Additional file 3 — Box 3. Accessibility of medical care. [file 2045-7022-2-12-S3.docx]

Box 3. Accessibility of medical care

| A | *“I’m still surprised – I was sure there would be sort of cross-contamination and stuff, which is quite bad, but there were certain meals I was eating and I was sort of…almost half-expecting an allergic reaction, which is kind of bad………. But then, I would only be taking that…having that kind of risk if I knew I was near a decent medical facility.” [1017, M, Moderate]* |
| --- | --- |
| B | *“My husband went to Kenya for two weeks and he took a group of older teenagers from our church, who I’ve worked with and done youth work with, and he…they went on like a mission thing, building a house, and I really desperately wanted to go, but I couldn’t because of the allergy. So, you know, decisions like that, I have to make in… You know, even…we were saying about a kind of risk assessment. The way you do risk assessment is that you look at kind of what are the chances of it happening, and then what’s the severity if it does happen, so even if the chances are small, if it does happen and I’m in a remote village in Kenya, there’s no way I’m going to help. That’s just not going to happen. It’s going to be a five or six hour journey.” [1116, F, Severe]* |
| C | *“If I’m ready for something, I have no problem, but it’s taken any spontaneity out of my life really. For example, we were in Cyprus and we were having an orange juice at this café, up a mountain in the middle of nowhere, in this tiny little village, and the lady said, “Oh, shall we have a typical Cypriot lunch here?” ……….. I would have really liked to have had a typical Cypriot lunch……… But I just couldn’t, for a fear of what might happen, and being so remote. So as I say, it’s taken all the spontaneity out.” [1029, F, Severe]* |
| D | *“We plan things quite meticulously from that perspective, even down to where’s the nearest hospital. Generally, we know where the nearest hospital is, so if there’s something up, we know where to go.” [1069, M, Severe]* |

**Key:** The study ID number is followed by the patient gender (F stands for female and M for male), followed by the severity of the participant’s worst allergic reaction to peanuts or tree nuts. "
