# Supplementary material for: The strategies that peanut and nut-allergic consumers employ to remain safe when travelling abroad
Source: Clin Transl Allergy. 2012 Jul 9;2:12. doi: 10.1186/2045-7022-2-12 (PMC3480958; doi:10.1186/2045-7022-2-12)
Supplement: Additional file 4 — Box 4. Unfamiliarity. [file 2045-7022-2-12-S4.docx]

Box 4. Unfamiliarity

| A | *“Well, we’re not that great at going on holiday [laughing], so not going is always an option! But, no, if…obviously, with my husband being Dutch, his family are still over in holiday, so the last few years, we’ve actually mainly gone over there, so him speaking the language helps a lot, and eating in places that he feels are safe.” [1042, F, Severe]* |
| --- | --- |
| B | *“Much more difficult. Not with as France much now, because we’ve been there so many times, but say I was to go to America or Indonesia or Africa, I would have no idea what to do, not the faintest idea, with the language. I could look at pictures [laughing], but that’s it. So I wouldn’t really know what to do.” [4008, M Severe]* |
| C | *“So that was really good. And then at restaurants, we have like translation cards, which are really good. There’s like two different ones. There’s one saying, “My child is having an anaphylactic shock – could you ring the ambulance?” in whatever language it is, and there’s one saying, “My child…” or “I have a nut allergy – can you cater for me? They’re allergic to this, this…” It also makes sure it says like “No oils with nuts in,” and it’s like really detailed.” [1008, F, Moderate]* |
| D | *“I always learn the word for “peanut” before I go.” [1161, F, Mild]* |
| E | *“I mean, we were talking about going to Thailand, and the nut allergy is playing a big part in that…because of the food there and its nut content, so we’re putting that one off a bit. We went to Turkey a few months ago, and I actually created some little flash cards [laughing] that said I’m allergic to nuts, and with a little picture of the nut with a red cross through, in Turkish.” [1060, M, Moderate]* |
| **F** | **And you have a [Name of adrenaline auto-injector] now that you carry around**? *“I carry two, and when I’m travelling, I carry six.”* **Do you?** *“Yeah. Well, in Europe, I would have only carried – I only carry two, but when I’ve travelled in Asia and when I was in Africa, I carry six, just because I don’t know if I’m going to be able to get any. It hasn’t occurred to me, but often reactions can come back a few hours later, so you might need to give yourself another shot of adrenaline. So em…yeah, I carry a lot of first aid, a pretty comprehensive kit when I’m travelling.” [4001, F, Severe]* |
| G | *“Medic Alert, you pay a certain…I think it’s £25 a year to be a member and you get a little bracelet – I don’t know where it is at the moment. What happens is, on this bracelet, and you’ve probably seen them – diabetics have them a lot – and it flips over and it actually says what your allergy – you’re given a number, so it’s not your name. It gives the number to phone in case of emergency and says what the allergy is.” [4013, F, Severe]* |

**Key:** The study ID number is followed by the patient gender (F stands for female and M for male), followed by the severity of the participant’s worst allergic reaction to peanuts or tree nuts. "
