# Supplementary material for: The strategies that peanut and nut-allergic consumers employ to remain safe when travelling abroad
Source: Clin Transl Allergy. 2012 Jul 9;2:12. doi: 10.1186/2045-7022-2-12 (PMC3480958; doi:10.1186/2045-7022-2-12)
Supplement: Additional file 5 — Box 5. Avoidance of high risk foods. [file 2045-7022-2-12-S5.docx]

Box 5. Avoidance of high risk foods

| A | *“Obviously, it’s a lot harder because of the packaging, but we normally go for English foods, just to be 100% sure.” [1016, F, Severe]* |
| --- | --- |
| B | *“I’ll stick to pizza or stuff that I know that’s not going to contain nuts.” [1023, M, Severe]* |
| C | *“I suppose it’s harder trying different cultures’ foods when you’re not so sure what’s in them. It probably sounds very boring to stick to shops that I know, even abroad, but it is I find the safest way” [1042, F, Severe]* |
| D | *“I prefer to prepare my own food when I go on holiday, unless you’re eating out in a restaurant, and in which case, again, I would only eat in restaurants that I would feel comfortable in really. So, abroad, especially if you don’t speak the language, it would be French and Italian restaurants, where I could have a pizza or something like that, or I go to the little local shop and buy the ingredients of what I know I’m going to cook, or whoever you’re going with, then you cook for them or they cook for you, in which case, you know what is going to be in the ingredients.” [4015, M, Severe]* |
| E | *“No. I think if – I mean, like I went to Turkey recently, and there was a lot of stuff there that I just don’t know, and I think, if I’m not 100% sure, I avoid it. I think you get a lot more nuts in desserts, so I really do not eat a lot of desserts, particularly abroad, because it’s just easier not to.” [4013, F, Severe]* |
| F | *“And also, when I’m with my parents, as I said, it’s different. Like even in a foreign country, it’s more risky, but it’s like… I went to Spain a couple of years ago with my friends, and that was worrying, because it’s in a different country, I don’t speak Spanish, and like, if we ate out, I was just like, oh, you know…it’s just worrying. But even in supermarkets, you just think how good is their labelling system…maybe…?” [1112, F, Severe]* |
| G | *“And…em, most of the holidays we go on are self-catering - I’ve never been all-inclusive - so I’ve always had a choice over what I eat. My parents don’t – if we’re abroad with them, they don’t buy peanuts anyway, and I’ll check the packaging and the back of everything because I know the foreign word for “peanut”, so… But I’ve found a lot more now, like France and Spain and things, the back of their packaging has always got some sort of allergy advice on, so it’s almost the same as being in England.” [1161, F, Mild]* |

**Key:** The study ID number is followed by the patient gender (F stands for female and M for male), followed by the severity of the participant’s worst allergic reaction to peanuts or tree nuts. "
